# Reverse protection assay: a tool to analyze transcriptional rates from individual promoters

**DOI:** 10.1186/1746-4811-7-47

**Published:** 2011-12-20

**Authors:** Yan O Zubo, Victor V Kusnetsov, Thomas Börner, Karsten Liere

**Affiliations:** 1 Institut für Biologie (Genetik), Humboldt-Universität zu Berlin, Chausseestrasse 117, D-10115 Berlin, Germany; 2 Timiriazev Institute of Plant Physiology, Russian Academy of Sciences, Botanicheskaya 35, Moscow, 127276 Russia; 3 Department of Biological Sciences, Dartmouth College, Hanover NH 03755, USA

**Keywords:** chloroplast, plastid transcription, promoters, RNA processing, run on transcription assay, RNase protection assay

## Abstract

Transcriptional activity of entire genes in chloroplasts is usually assayed by run-on analyses. To determine not only the overall intensity of transcription of a gene, but also the rate of transcription from a particular promoter, we created the Reverse RNase Protection Assay (RePro): in-organello run-on transcription coupled to RNase protection to define distinct transcript ends during transcription. We demonstrate successful application of RePro in plastid promoter analysis and transcript 3' end processing.

## Background

Today's molecular biology employs several methods to determine the steady-state levels of RNAs, such as Northern- and dot-blot analysis, ribonuclease protection assay (RPA), reverse transcription-PCR (RT-PCR), and quantitative real-time PCR. These methods differ in their sensitivity and information content. For example, while RT-PCR allows quantification of RNA molecules of different lengths, RPA permits determination of the relative amount of transcripts with distinct 5'- and/or 3'-ends [1]. In general, RPA is based on hybridization of a labeled, single-stranded antisense RNA probe to the target RNA. Subsequent incubation with an RNases mix degrades those RNA molecules that do not form double-stranded hybrids. Final inactivation of RNases and precipitation of the protected RNA hybrids is followed by electrophoretic analyses, which reveals the presence, size, and relative level of RNA that was protected by the antisense probe [1]. The RPA method has been modified to serve for different tasks, such as measuring the radioactive signals by scintillation counting [RiPPA method; [2]], and replacing radiolabeled with biotinylated probes [3]. Adding constitutively transcribed or *in vitro *synthesized RNA as an internal standard during RNA isolation allows quantification of the RNA analyzed by RPA [4,5].

However, the overall RNA content reflects a balance between the synthesis and degradation of transcripts [6]. The aforementioned methods only identify RNA steady-state levels. To directly evaluate the rate of individual gene transcription, run-on transcription is utilized: transcripts are labeled by adding radio-nucleotides during a brief time of incubation and subsequently analyzed by dot-blot hybridization [1]. This technique applies to all genetic compartments such as bacterial cells, nuclei, mitochondria, and chloroplasts. However, this method also has some limitations. The double-stranded probes usually used for dot-blots in run-on assays are able to hybridize with both sense and antisense transcripts. Interfering transcription initiated by adjacent promoters on the opposite strand may disturb the results obtained by this method. This is especially significant for transcription of bacterial and chloroplast genes, where a great amount of antisense transcripts are generated [e.g., [7,8]]. Furthermore, the size of transcripts analyzed and the corresponding promoter(s) remain unknown.

Generally, plastids possess a rather inefficient transcriptional termination system. Although inverted repeat sequences, which can fold into stem-loop structures similar to bacterial terminators, are often found at 3' ends of plastid transcripts, they do not serve as terminators, but rather function as RNA 3' end processing and maturation signals [9-11]. Leaky termination might therefore be one of the reasons for the observed dual transcription of certain genes both from their own promoter and as part of a polycistronic operon [7,12-19].

We have previously shown that synthetic cytokinin 6-benzyladenine (BA) enhanced transcription rates of several chloroplast genes in barley. Among the most affected was the *rrn16 *operon [20]. To date, the only promoter known to drive transcription of the *rrn16 *operon in barley is a sole PEP promoter [P*rrn16*-118/-119; [21], PEP, plastid-encoded plastid RNA polymerase; reviewed in [22,23]]. However, directly upstream of the *rrn16 *operon, the gene for tRNA^Val ^(GAC) is located (*Hordeum vulgare *plastid genome: GenBank EF115541). To investigate if the target of cytokinin action to activate *rrn16 *gene expression is increased transcription initiation from P*rrn16*-118/-119, we developed a novel method, which couples transcription and RNA mapping, the Reverse RNA Protection assay (RePro). Essentially, RePro is a combined technique of in-organello run-on transcription in the presence of radiolabeled nucleotides and an RNase Protection Assay (RPA) using unlabeled RNA probes, with the advantages of both of these methods. In principle, RePro allows the accurate analysis of the rate of transcription from individual promoters, which is a crucial step in studies of regulation of gene expression.

## Results and discussion

### Mapping of 5'-ends of *rrn16*, *rbcL*, and *psbA *transcipts by RePro

**Figure 1:**
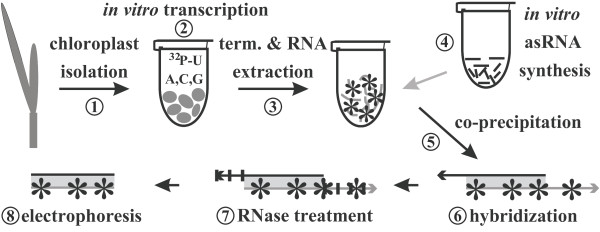
**Scheme of Reverse RNase Protection Assay (RePro) procedure**. (1) Isolated organelles (after quantification) are immediately used for (2) *in vitro *transcription in the presence of labeled nucleotides (indicated by A, C, G, U). During *in vitro *transcription (10 min usually), newly synthesized RNAs that arise from either transcript initiation or elongation of RNAs initiated before chloroplast isolation are labeled by incorporation of radioactive nucleotides (asterisks). (3) The reaction is terminated by the addition of stop buffer and the RNA is subsequently isolated. (4) The required amount of *in vitro *generated asRNA (about 0.1 μg) is added to the chloroplast RNA and both RNAs are co-precipitated (5). The RNA pellet obtained is dissolved in hybridization buffer and hybridized at 64°C (6). (7) To remove none-hybridized, single-stranded RNAs RNase treatment is performed and the samples are subsequently analyzed on 4% polyacrylamide/8 M urea gels (8) according to the standard RNase Protection Assay protocol (RPA).

The reverse RNA protection assay (RePro) is technically divided into three stages: (1) chloroplast isolation, (2) run-on transcription in presence of [^32^P]-UTP and RNA isolation from the reaction mixture, and (3) RNase protection with unlabeled anti-sense RNA (asRNA) with subsequent analysis of the protected double-stranded RNA fragments by gel electrophoresis (Figure 1).

**Figure 2:**
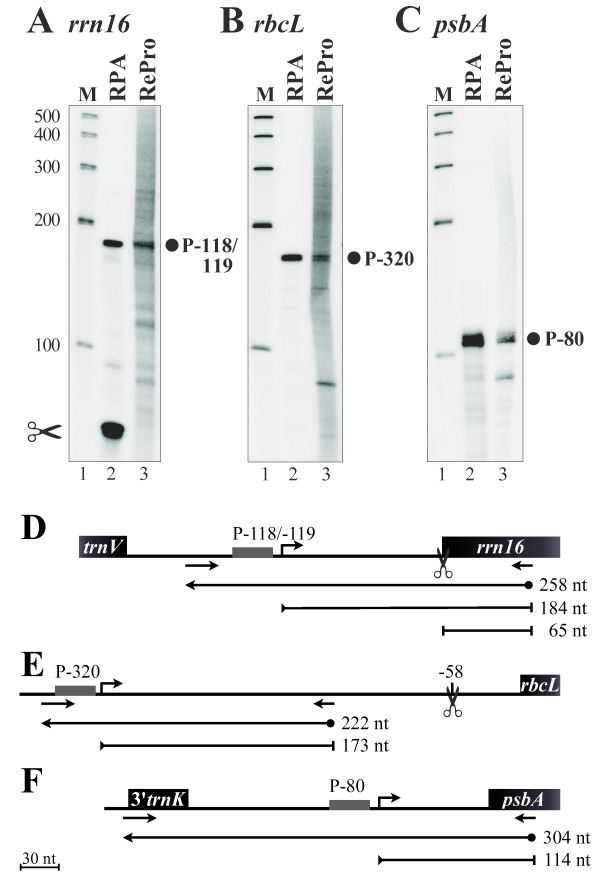
**Validation of the Reverse RNase Protection Assay**. The 5'-ends of *rrn16 *(**A**), *rbcL *(**B**), and *psbA *(**C**) transcripts were analyzed by RNase Protection Assay (RPA, lanes 2) and Reverse RNase Protection Assay (RePro, lanes 3). Mapped transcription initiation sites (filled circles) are identified by their distance between the transcription initiation site and the first nucleotide of the mature RNA (*rrn16*) or the translation initiation codon (*rbcL*, *psbA*) in nucleotides. Molecular weight marker in nt is provided on the sides (M, lanes 1). The positions of probes, primers, and protected fragments are shown in (**D**), (**E**), and (**F**) for *rrn16*, *rbcL*, and *psbA*, respectively. Scissor symbols denote RNA processing sites. Black boxes denote coding regions; small arrows indicate primers; grey boxes and angled arrows indicate promoters and transcription initiation sites; ball-ended arrows indicate complementary RNA probes; bar-ended lines denote protected RNA fragments. Respective sizes are given in nucleotides (nt). Note that the RNA probe sizes include 6 nt added to the transcripts from the T7 promoter. A scale is provided on the bottom left.

To validate the RePro concept, known transcript 5'-ends of the *rrn16*, *rbcL*, and *psbA *genes corresponding to the PEP promoters from barley P*rrn16*-118/-119 [21], P*rbcL*-320 [24], and P*psbA*-80 [25] were simultaneously analyzed both with the novel run-on based RePro (Figure 2, lanes 3) and the steady-state level based RPA as a control (Figure 2, lanes 2). To ensure sufficient amounts of primary transcripts, one-day-old barley seedlings where used to analyze *rrn16 *5'-ends, while 6-day-old leaves were used to analyze the transcript 5'-ends of the *psbA *and *rbcL *genes. The RPA assay, after addition of labeled *rrn16 *asRNA probe (258 nt, Figure 2D), gave rise to protected RNA fragments of 65 nt, which correspond to the processed form, and 184 nt, which correspond to the primary transcript (Figure 2A, lane 2; Figure 2D). Similarly, hybridization of the unlabeled *rrn16 *asRNA probe (258 nt, Figure 2D) to the RNA generated by run-on transcription in presence of [^32^P]-UTP as well identified a 184 nt fragment corresponding to the primary transcript in the RePro assay (Figure 2A, lane 3). However, no processed transcript 5'-ends were detected. Similarly, protected fragments of 173 and 114 bp were obtained in both the RPA (Figure 2B and 2C, lanes 2) and RePro (Figure 2B and 2C, lanes 3) corresponding to the primary transcripts of *rbcL *and *psbA *mRNAs, respectively. To test RePro for being capable of detecting less strongly initiated transcripts we analyzed transcriptional initiation of the *trnM*-CAU PEP promoter [26]. Although weakly, initiated *trnM *transcripts were indeed detectable (data not shown). Therefore, the novel technique of reverse RNA protection assay is indeed capable of detecting 5'-ends of newly synthesized transcripts attributed to a certain promoter, which were generated by run-on transcription.

### Origin of non-specific background signals in the RePro

**Figure 3:**
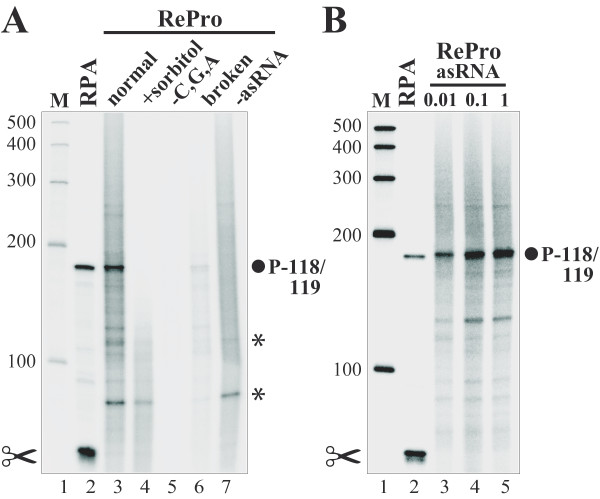
**Analyses of RePro reaction conditions**. (**A**) The 5'-ends of *rrn16 *transcripts were analyzed by RNase Protection Assay (RPA, lane 2) and Reverse RNase Protection Assay under different conditions (RePro, lanes 3-7). The RePro conditions tested were a full assay (normal, lane 3); addition of sorbitol to a final concentration of 0.33 M (+sorbitol, lane 4); *in vitro *transcription performed in the presence of [^32^P]-UTP but without GTP, CTP, and ATP (-C, G, A, lane 5); broken chloroplasts (broken, lane 6); a control for self-protection of endogenous transcripts without the addition of asRNA (-asRNA, lane 7). (**B**) The effect of the amount of asRNA in RePro on the signal intensity. 5'-ends of *rrn16 *transcripts were analyzed by RNase Protection Assay (RPA, lane 2) and Reverse RNase Protection Assay with increasing amounts of unlabeled antisense RNA (RePro): 0.01 μg (lane 3), 0.1 μg (lane 4), 1 μg (lane 5). Mapped *rrn16 *transcription initiation site (filled circle) is identified by its distance between the transcription initiation site and the first nucleotide of the mature rRNA. The scissors symbol denotes a RNA processing site. Signals of labeled chloroplast transcripts, which are protected against RNase treatment, are marked with asterisks. Note that patterns of additional bands may differ between individual RNA samples. Molecular weight marker in nt is provided on the side (M, lanes 1).

In addition to specific signals, some additional bands were detected in RePro assays (e.g., ~80 bp and 110-130 bp fragments; Figure 3A, lane 3, asterisks). To understand the nature of these bands, we tested the system for the possibility of double-stranded RNAs generated by endogenous transcripts, which thereby are protected against RNase treatment. To this end, a RePro assay was performed as outlined before, however, no gene specific unlabeled asRNA was added. Still, these non-specific bands appeared in the absence of asRNA as well (Figure 3A, lane 7, asterisks), thus indicating that certain endogenous chloroplast transcripts are protected against RNase treatment after labeling by run-on transcription. Note that the number and intensity of such additional bands may differ between individual RNA isolations and samples. Plastid genes are located on both DNA strands of the plastome [27]. Thus, some transcripts could originate from the same region, however, initiated from different DNA strands resulting in double-stranded RNase-protected transcripts. Furthermore, plastid RNAs such as some tRNAs [28] may form highly stable secondary structures, which lead to intra-molecular protected RNA fragments. Interestingly, the intensity of the ~80 bp protected fragment varied depending on the age of the leaves. In samples of older, 9-day-old leaves (i.e., Figure 2A, 3B, and 6A), the signal of the 80-nt unspecific fragment was less intense in comparison to those of 6-day-old leaves (Figure 3A and 5B) suggesting that the amount of the transcript which gives rise to this protected RNA fragment is declining during leaf development.

To investigate the influence of the integrity of the chloroplasts, RePro with chloroplasts isolated from the Percoll gradient as intact or broken were carried out (Figure 3A). Since the broken chloroplasts displayed a much lower transcriptional activity (Figure 3A, lane 6), only intact organelles should be used as a source for run-on transcription (Figure 3A, lane 3). However, the run-on transcription reaction is performed in a chloroplast lysate, because of a sharp drop in the osmotic strength when the isolation buffer is replaced by transcription buffer. To test if the chloroplast integrity is further required, we performed RePro assays where sorbitol was added to transcription buffer to the final concentration of 0.33 M to avoid osmotic lysis. Evidently, lysis of the chloroplasts before or during *in vitro *transcription is necessary to allow fast and easy accessibility of exogenously added (radiolabeled) nucleotides to the plastid transcription system [lanes 4 and 5; [29]].

### Optimization of the unlabeled asRNA amounts in RePro

The amount of labeled asRNA probes in conventional RPAs does usually not exceed 1 ng [1]. To determine the best amount of unlabeled asRNA, RePro assays with increasing amounts of unlabeled asRNA were performed (Figure 3B). An increase from 0.01 to 0.1 μg of the unlabeled asRNA enhanced the signal by approximately 30% (Figure 3B, lanes 3, 4). A further increase in the asRNA amount to 1 μg did not result in further signal enhancement (Figure 3B, lane 5), which indicates saturation of the system. The asRNA should never be the limiting factor for hybridization with the corresponding chloroplast transcripts labeled by run-on transcription. Otherwise, it would not be possible to compare distinct transcriptional rates. Therefore, we typically added 0.1 μg of unlabeled asRNA to [^32^P]-UTP-labeled chloroplast RNA isolated from run-on transcription with about 5 × 10^8 ^chloroplasts.

### RePro exhibits similar increases in signal over time as run-on analysis

Unlike the run-on assay, RePro does not fully reflect the accumulation of full-length and/or elongated but processed transcripts, but of relatively short 5'-fragments defined by the design of the asRNA for the protection assay. In our experiments, a 184-nt fragment of the *rrn16 *transcript 5'-end was protected. Therefore, the effect of transcript elongation on the detectable amount of freshly transcribed RNAs may be limited. Moreover, it is generally believed that incorporation of [^32^P]-label in run-on assays only occurs during elongation of already initiated transcripts [29,30]. RNA synthesis is greatly reduced after chloroplast isolation (performed at 4°C) and is restored under temperature conditions of about 25°C. Supposedly, the rate of the reaction during the first 10 min reflects the intensity of RNA transcription. Thereafter, RNA degradation and/or accumulation start to influence the observed amounts of labeled transcripts [29]. Transcription due to new initiation by plastid RNA polymerases during the run-on process occurs slower than *in planta *is therefore considered insignificant [29].

**Figure 4:**
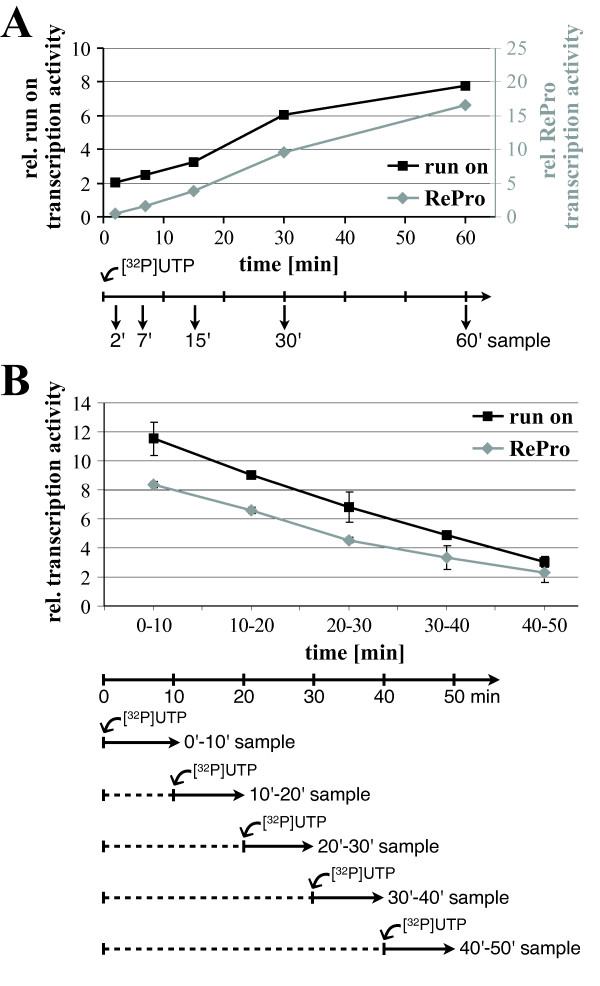
**(A) Comparison of the incorporation of labeled nucleotides of *rrn16 *gene transcription over time in run-on (black line, y-axis on the left) and RePro (grey line, y-axis on the right) systems, measured after 2, 7, 15, 30, and 60 min of reaction time**. (**B**) Decline of transcriptional activity in isolated chloroplasts. The amount of labeled *rrn16 *transcripts was analyzed in run-on (black line) and RePro (grey line) systems by pulsing the reaction by adding [^32^P]-UTP during indicated intervals (0 - 10, 10 - 20, 20 - 30, 30 - 40, and 40 - 50 min). A schematic representation of the timing of [^32^P]-UTP addition and RNA sampling is given below each graph.

To compare the kinetics of run-on assay and RePro over time, we performed *in vitro *transcription with isolated chloroplasts in the presence of [^32^P]-UTP. An aliquot was removed at time points of 2, 7, 15, 30, and 60 min and the RNA isolated. One-third of the RNA was used for hybridization with spotted *rrn16 *amplicons (Figure 4A, run-on) and two-thirds were used for a protection assay using unlabeled anti-sense transcripts of the 5' region of the *rrn16 *gene (Figure 4A, RePro). Both methods displayed a similar time curve of transcript accumulation. After an increase in the first 7 min, transcript accumulation showed an even higher rate of increase until 30 min, which to a lesser degree still increased until 60 min of *in vitro *transcription. Therefore, initiation of transcription seems to play a significant role in determining RNA levels and seems to be detectable with both methods. Furthermore, these data suggest that the reverse protection assay is capable to capture the kinetics of transcription from a single promoter as the run-on assay does for an entire gene. Interestingly, the accumulation of labeled transcripts increased slower between 30 and 60 min of incubation when detected by run-on than in case of RePro analysis. After this period of time the previously *in vivo *initiated and partly elongated transcripts may have been finished and no longer contribute to the increase of detectable, labeled RNAs. However, since RePro by design detects initiated transcripts rather than elongated RNAs located further downstream of the probe, new initiation adds more constantly to the pool of labeled transcripts detected by RePro. This further suggests that elongation is better assessed by run-on analyses, while RePro detects newly initiated transcripts.

### Decline of transcriptional activity in isolated chloroplasts

To evaluate the robustness of the plastid *in vitro *transcription system over a time course and to compare the analytical methods of run-on and RePro, we pulsed *in vitro *transcription assays by successively adding [^32^P]-UTP during a 50 min time course (Figure 4B). To measure the rate of transcription during first 10 min, we added [^32^P]-UTP at the moment of the reaction start. To measure the rate of transcription during subsequent 10-min intervals, we firstly performed the transcription reaction for 10, 20, 30, or 40 minutes with unlabeled nucleotides, followed by an additional incubation of 10 min with added [^32^P]-UTP. RNA isolated from each sample was processed as described for run-on (black graph) and RePro analyses (grey graph). Both methods detected a comparable, nearly linear decline of about 80% of the transcriptional activity in isolated chloroplasts during the 50 min of incubation. Interestingly, a less pronounced slope of the transcriptional activity was observed after 30 min (20 min without label, followed by 10 min with [^32^P]-UTP), a point of time which showed increased transcript accumulation when incubated the entire period of 30 min with [^32^P]-UTP (Figure 4A). This suggests that stabilization of *rrn16 *RNAs generated by run-on transcription significantly occurs after 20 to 30 min of reaction time. However, during further incubation up to 60 min, the RNA stability seemed to decline again. Since only label incorporated into the protected RNA 5'-ends of 184 nt contributes to the RePro signal, it might be possible that initiation of new transcripts is slower than the elongation of transcripts initiated *in planta*, which are still detectable by run-on but not with RePro.

### Pulse-chase experiments to determine the stability of synthesized RNAs

**Figure 5:**
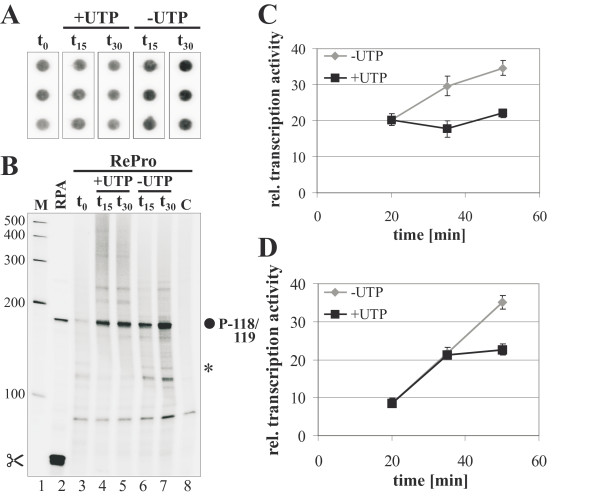
**Analyses of degradation processes on labeled transcript accumulation by pulse-chase labeling in run-on (A, C) and RePro systems (B, D)**. After 20-min transcription in the presence of [^32^P]-UTP (t_0_), unlabelled UTP was added to the reaction mixture (+UTP), and the reaction was let run further for 15 (t_15_) or 30 min (t_30_). Control reactions without addition of unlabeled UTP were performed in parallel (-UTP). (**A**) Dot-blot analysis of a typical run-on reaction hybridized to *rrn16 *DNA fragments spotted in triplicates. (**B**) 5'-ends of *rrn16 *transcripts were analyzed by RNase Protection Assay (RPA, lane 2) and Reverse RNase Protection Assay at indicated points of time with or without the addition of unlabeled UTP (lanes 3-7). A control for self-protection of labeled *rrn16 *transcripts after 35 min of reaction time, however, without the addition of unlabeled asRNA is shown in lane 8. Mapped *rrn16 *transcription initiation site (filled circle) is identified by its distance between the transcription initiation site and the first nucleotide of the mature rRNA. Molecular weight marker in nt is provided on the side (M, lane 1). An additional signal of about 130 bp of an potentially elongation arrested transcript is marked by an asterisk. Data shown for the representative autoradiograms may slightly differ from the averages of multiple experiments shown in (**C **and **D**). (**C**) Relative rates of transcription (× 1000) for the *rrn16 *gene as determined by run-on analyses in (**A**). (**D**) Relative rates of transcription (× 1000) for the *rrn16 *gene as determined by RePro in (**B**).

Degradation processes may have an influence on the detectable amount of the transcripts in both run-on and RePro assays, especially after 30 min of the reaction. To further investigate if RNA degradation might play a role in both run-on and RePro detection systems under prolonged reaction time conditions pulse-chase experiments were carried out (Figure 5). *In vitro *transcription with isolated chloroplasts was performed for 20 min in the presence of [^32^P]-UTP. Subsequently, unlabeled UTP was added to a final concentration of 1.11 mM, and the reaction was allowed to continue for 15 or 30 min. Samples were taken at the moment of adding unlabeled UTP (t_0 _= 20 min), after 15 min (t_15 _= 35 min) and 30 min (t_30 _= 50 min). The addition of unlabeled UTP was omitted in control samples taken at the same time points. The isolated RNA was analyzed in run-on (Figure 5A and 5C) and RePro (Figure 5B and 5D) experiments with *rrn16 *gene specific probes. Quantification of the signals obtained by run-on analysis showed no significant change of labeled *rrn16 *transcript levels after addition of unlabeled UTP, while further incubation in the presence of [^32^P]-UTP increased the detectable amounts about 1.7× (Figure 5C). Similarly, increasing accumulation of *rrn16 *5'-ends was detected during prolonged incubation in the presence of [^32^P]-UTP (Figure 5D, gray graph). In contrast to the run-on analyses, the RePro assay showed an increase of *rrn16 *5'-end levels until 15 min after UTP addition comparable to the control. However, the labeled *rrn16 *transcript levels then did not change during further incubation (Figure 5D, black graph). The fact that neither run-on nor RePro showed a decline of labeled *rrn16 *transcript levels after the addition of unlabeled UTP, nonetheless argues for the absence of *rrn16 *transcript degradation in our *in vitro *transcription system. Therefore, the gradual reduction of transcriptional activity in this system may be due to termination of elongation of transcripts, which were initiated before plastid isolation, depletion of components, and decreasing robustness of enzymes involved. However, *rrn16 *by itself is a stable transcript. These results may be different with other, less stable plastid genes.

The surprising effect of still rising amounts of labeled *rrn16 *transcripts 15 min after adding the unlabeled UTP (Figure 5B and 5D), which was not observed using the conventional run-on technique, might partly be explained by a transcriptional arrest caused by UTP deficiency in the *in vitro *transcription system. Adding unlabeled UTP might remove the elongation arrest and RNA synthesis resumes. These partially labeled transcripts are now fully detectable by the antisense RNA probe and may therefore more substantially add to the weak reverse protection signal as compared to the solid phase hybridization signal in standard run-on analyses. Indeed, an additional signal of about 130 bp appears in samples without the addition of unlabeled UTP, but is absent from samples where unlabeled UTP was added (Figure 5B, asterisk). In run-on assays, however, such an increase of the *rrn16 *transcript signal was not observed 15 min after adding unlabeled UTP (Figure 5A and 5C). Two reasons may account for this: firstly, the length of transcripts is unimportant for hybridization to the probe on the dot blot; secondly, the amount of labeled transcripts then being able to hybridize after lifting the arrest is too small to contribute significantly to the entire signal.

### RePro assays are capable of both determining transcription rates of individual promoters and monitoring RNA processing

**Figure 6:**
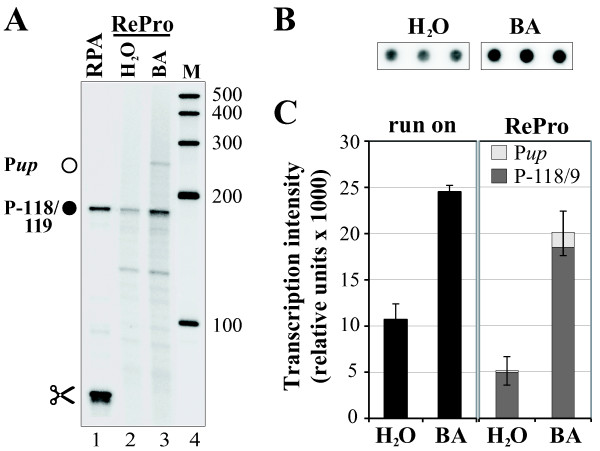
**Regulation of transcription from the promoter P*rrn16*-118/-119 by cytokinin**. (**A**) Analysis of 5'-ends of *rrn16 *transcripts by RNase Protection Assay (RPA, lane 1) and Reverse RNase Protection Assay (RePro, lanes 2, 3). RNA used for RePro was isolated from leaf chloroplasts, which were incubated either on water (H_2_O, lane 2) or cytokinin (BA, lane 3). Mapped *rrn16 *transcription initiation site (filled circle) is identified by its distance between the transcription initiation site and the first nucleotide of the mature rRNA. P*up *(open circle) denotes a signal of the size of the fully protected RNA probe possibly representing transcription initiation from an upstream promoter. Molecular weight marker in nt is provided on the side (M, lane 4). The scissors symbol denotes a RNA processing site. (**B**) Dot-blot analysis of a typical run-on reaction of chloroplasts isolated from leaves detached from 9-day-old barley plants which were incubated on water (H_2_O) or cytokinin (BA) hybridized to *rrn16 *DNA fragments spotted in triplicates. (**C**) Relative rates of transcription for the *rrn16 *gene as determined by run-on analyses in (**A**) and RePro in (**B**). Data shown for the representative autoradiograms in (**A**) and (**B**) may slightly differ from the averages of multiple experiments shown in (**C**).

To date, a single promoter is known to drive transcription of the *rrn16 *operon in barley [P*rrn16*-118/-119; [21]]. However, in some RPA and RePro experiments we observed a weak signal corresponding to a fully protected probe (258 nt, Figure 2D), which suggests *rrn16 *transcripts originating further upstream (e.g., Figure 3B and 5B; data not shown for RPA assays). Furthermore, transcripts of this gene still accumulated in the barley *albostrians *mutant lacking transcription from P*rrn16*-118/-119 suggesting read-through from transcripts originating further upstream [21]. Transcription of the plastid *rrn16 *gene is activated by the plant hormone cytokinin (BA) as has been shown by run-on analyses [Figure 6B; [20]]. To study to what extent the read-through from upstream contributes to this transcriptional activation, we used RePro to analyze transcriptional rates from the PEP promoter P*rrn16*-118/-119 in chloroplasts extracted from the apical parts of leaves of 9-day-old plants treated with either water or BA (Figure 6A). In comparison to the water control (Figure 6A, lane 2), transcription of *rrn16 *from P*rrn16*-118/-119 was activated approximately threefold by BA (Figure 6A, lane 3), which was about equivalent to the induction observed by run-on analysis (Figure 6C). Furthermore, a ~260-nt signal corresponding to a fully protected probe was detectable after BA treatment (P*up*; Figure 6A, lane 3), which contributed to about 10% of the total transcriptional activity (Figure 6C; panel RePro). In the water control, read-through from P*up *contributed only about 0.5%. Thus, treatment by cytokinin also activates transcription upstream of P*rrn16*-118/-119, which significantly contributes to the overall transcriptional activity observed in run-on assays. Furthermore, we could show that it is indeed possible to determine transcriptional rates from individual promoters using RePro assays.

**Figure 7:**
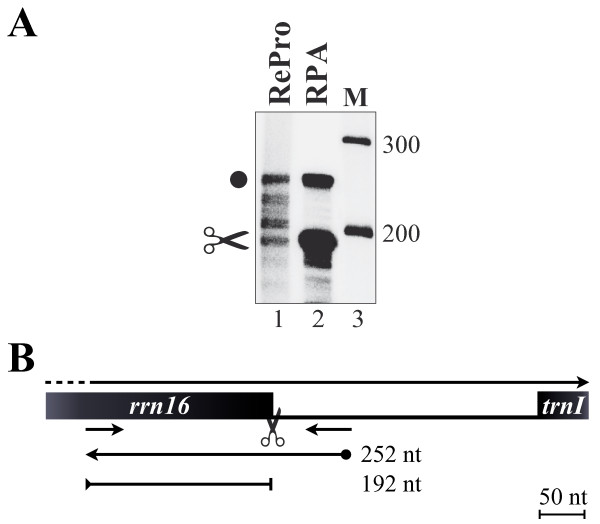
**Monitoring of *rrn16 *3'-end processing**. (**A**) Analysis of 3'-ends of *rrn16 *transcripts by Reverse RNase Protection Assay (RePro, lane 1) and RNase Protection Assay (RPA, lane 2). Molecular weight marker in nt is provided on the side (M, lane 3). The positions of probes, primers, and protected fragments are shown in (**B**). Scissor symbols denote RNA processing sites. Black boxes denote genes; small arrows indicate primers; the ball-ended arrow indicates the complementary RNA probe and the fully protected RNA fragment; the bar-ended line denotes the protected RNA fragment representing the processed mature *rrn16 *3'-end. Respective sizes are given in nucleotides (nt).

Generally, plastids possess a rather inefficient transcriptional termination system and read-through transcription has been reported for several genes and operons [7,9-19]. The *rrn16 *gene is the first gene to be transcribed within an operon, which further includes several tRNA and rRNA genes [31,32]. To test, if RePro is able to monitor RNA processing of the primary transcript during transcription, we performed RePro and RPA assays on the *rrn16 *3' end (Figure 7). After performing RPA analyses, protected RNA fragments of 192 nt and 252 nt were obtained (Figure 7A, lane 2), which correspond to the processed and non-processed 16S rRNA 3' ends, respectively (Figure 7B). Similarly, these bands were also observed using RePro (lane 1). In comparison to RPA analyses, however, several additional RNA fragments were detectable in RePro assays, which most likely represent intermediate maturation products. Therefore, RePro assays are not only capable of testing RNA synthesis from distinct promoters, but also the synthesis of new transcripts and their processing.

## Conclusion

We developed a novel method, the Reverse RNA Protection assay (RePro), which allows the accurate analysis of distinct transcript 5'- and 3'-ends during the transcriptional process by combining run-on and RNase protection assays. Using RePro, we were able to show that about 10% of the transcriptional activation of the *rrn16 *gene by cytokinin comes from read-through transcription initiated upstream of its P*rrn*-118/-119 promoter. Furthermore, although the important initial step of RePro, the run-on assay, is thought to be a system of transcription elongation, we clearly observed substantial contribution of *in vitro *transcription initiation. We additionally demonstrated that RNA degradation processes did not affect the results obtained by RePro.

In general, application of RePro allows an additional analysis of gene transcription, i.e., testing transcription from individual already known promoters and processing of freshly generated transcripts. The effects of transcription from other promoters of the gene and nonspecifically transcribed sense or antisense RNA synthesis are completely excluded. RePro reflects changes occurring at the level of *in vitro *transcription initiation more adequately.

## Methods

### Plant growing and benzyladenine treatment

Experiments were performed with primary leaves of 6- to 9-day-old barley plants (*Hordeum vulgare *L., cv. Luch), which were grown in soil at 20°C under illumination of 270 μmol m^-2 ^s^-1 ^from luminescent tubes (Lamp Master HPI-T Plus 400 W E40, Philips) under a 16-h photoperiod. For experiments on transcriptional regulation by cytokinin, primary leaves were detached from 9-day-old plants and incubated on filter paper moistened with water under continuous illumination of 270 μmol m^-2 ^s^-1 ^for 24 h. Subsequently, leaves were transferred to water or 2.2 × 10^-5 ^M BA and kept for 3 h under the same light conditions.

### Run-on - chloroplast isolation

Apical segments (2 cm in length) of the first leaves detached from 9-day-old barley plants were used for chloroplast isolation, because preliminary experiments have demonstrated that this leaf part is most sensitive to cytokinin treatment [20]. Leaf segments (10 g) were homogenized in 80 ml of buffer A (0.33 M sorbitol, 50 mM Tricine, pH 8.0, 2 mM EDTA, and 5 mM β-mercaptoethanol). The homogenate was squeezed through Miracloth (Calbiochem, San Diego, CA, USA) and centrifuged at 2700 *g *for 6 min. The pellet was resuspended in 1.5 ml of buffer A and fractionated in a 40/70% discontinuous Percoll gradient by centrifugation at 4000 *g *for 30 min. Intact chloroplasts were collected at the interface between 40 and 70% Percoll, washed in buffer A, and resuspended in 0.5 to 1 ml of buffer B (50 mM Tris-HCl, pH 7.0, 10 mM MgCl_2_, 10 mM KCl, and 4 mM β-mercaptoethanol). The number of chloroplasts in the samples was determined by counting the organelles under a light microscope using a Fuchs-Rosenthal hemocytometer [33]. Chloroplasts (5 × 10^8^) were precipitated by centrifugation, resuspended in 45 μl of buffer D (50 mM Tris-HCI, pH 7.0, 10 mM MgCl_2_, 10 mM KCl, 4 mM β-mercaptoethanol), and used for run-on transcription. All procedures were performed at 4°C.

### Run-on - *in vitro *transcription reaction

*In vitro *transcription reaction with 5 × 10^8 ^lysed plastids was carried out in a 100 μl volume by the method of Mullet and Klein [6] modified according to Zubo et al. [34]. Transcription was performed for 10 min at 25°C in buffer containing 50 mM Tris-HCl, pH 8.0, 10 mM MgCl_2_, 0.2 mM CTP, GTP, ATP, 0.01 mM UTP, 50 μCi of [α-^32^P]-UTP (≈3000 Ci/mmol, Amersham, UK), 20 U of RNasin (Fermentas, Lithuania), and 10 mM β-mercaptoethanol. The reaction was stopped by the addition of an equal volume of stop buffer (50 mM Tris-HCl, pH 8.0, 25 mM EDTA, and 5% sarcosyl). [^32^P]-labeled transcripts were isolated from chloroplasts as described by Guadino and Pikaard [35]. Reaction products were extracted twice with phenol/chloroform and once with chloroform. RNA was precipitated by an equal volume of isopropanol in the presence of 0.1 volume of 3 M sodium acetate, pH 6.0, and 20 μg of tRNA.

Blotting of plastid gene probes, their positions, and hybridization conditions in conventional run-on experiments were carried out as described in Zubo et al. [20]. Radioactive signals of dot blot assays were detected and quantified using a Molecular Imager FX and the Quantity One software (Bio-Rad, USA). The results, termed 'relative transcription activity', of three independent biological with two technical replicates were analyzed and visualized using Microsoft Excel (see Figures 4, 5, and 6).

### RNAse protection reaction

The assay was performed according Sambrook et al. [1], however, with slight changes. Performed as control reactions, RNase protection assays contained 1 μg (*rrn16*) or 5 μg (*psbA *and *rbcL*) of chloroplast RNA and [^32^P]-UTP-labeled of the respective asRNA (about 3 × 10^5 ^cpm, MEGAscript T7 kit, Ambion), which were subsequently co-precipitated with ethanol, dissolved in 30 μl hybridization buffer (40 mM PIPES, pH 6.8, 1 mM EDTA, pH 8.0, 0.4 M NaCI, 80% deionized formamide), denatured (5 min 90°C), and incubated at 60°C over night. After adding 300 μl RNase digestion buffer (300 mM NaCl, 10 mM Tris-HCl, pH 7.4, 5 mM EDTA, pH 7.5) and 5 μl of a RNaseA/RNaseT1 Mix (Fermentas, Lithuania; 2 mg/ml of RNase A, 5000 U/ml of RNase T1), digestion of single-strand RNAs was performed by incubation at 30°C for 1 hour. The digestion was stopped by adding 20 μl of 10% SDS and 10 μl of 10 mg/ml proteinase K (Fermentas, Lithuania) and further incubation at 37°C for 30 min. The RNAs were treated with phenol/chloroform, ethanol precipitated in the presence of 20 μg of carrier yeast tRNA (Invitrogen), and resuspended in 10 μl of gel-loading buffer (95% deionized formamide, 0.025% bromophenol blue, 0.025% xylene cyanol, 5 mM EDTA, pH 8.0, 0.025% SDS). Protected RNA fragments were fractionated in 4% polyacrylamide/8 M urea gels in Tris-borate buffer, pH 7.7. After exposure, the gels were analyzed with a PhosphoImaging system and specific bands and signals quantified using the complementary software (Quantity One, BioRad). The results of three independent biological with two technical replicates were analyzed and visualized using Microsoft Excel.

In case of the reverse RNase protection assay, the [^32^P]-UTP-labeled RNA synthesized in a run-on experiment *in vitro *and unlabeled asRNA (from 0.01 to 1 μg) were co-precipitated at -20°C for 1 h and subsequently handled as outlined for the RNase protection assay.

The length of protected RNA fragments was determined using the RNA Century™ Marker Templates (Ambion, USA) according to the manufacturer's protocol.

### *In vitro *antisense RNA synthesis

Synthesis of antisense RNA (asRNA) was performed using the MEGAscript T7 Kit (Ambion, USA) according to the manufacturer's protocol using PCR fragments comprising the promoter region of the gene studied as templates. Each reverse primer contained the sequence of the T7 promoter (lowercase letters). The following primers were used (for positions see also Figures 2 and 7):

rrn16-for CGAGCGAACGAGAATGGATAAGAG, rrn16-rev cagagatgcataatacgactcactata**g**ggagaCGACTTGCATGTGTTA; rrn16-e-up GTAATCGCCGGTCAGCCATAC, rrn16-e-low cagagatgcataatacgactcactatagggagaTGAAGAAGTGTCAAACC; psbA-for CCGACTAGTTCCGGGTTCGAG, psbA-rev cagagatgcataatacgactcactata**g**ggagaTTGTACTTTCGCGTC; rbcL-for TAATTTGGGTTGCGCTATACCTATCA, rbcL-rev cagagatgcataatacgactcactata**g**ggagaTTGAGGGCATGCT. After synthesis and treatment with DNase I, the [^32^P]-UTP-labeled asRNA was purified by electrophoresis in a denaturing polyacrylamide gel to remove shorter than full-length RNAs as well as the DNA template. RNA was eluted from the gel by gel fragment incubation in buffer containing 1 mM EDTA and 0.2% SDS (350 μl per gel fragment) overnight at 37°C with constant weak shaking. In the case of unlabeled asRNA, the procedure of electrophoretic purification was omitted. Firstly, no additional bands are seen after electrophoresis if the DNA template protects unlabeled asRNA, since the chloroplast RNA is labeled but not the asRNA. Secondly, unlabeled RNA is more stable because of the absence of its self-degradation by radioactive radiation.

## Competing interests

The authors declare that they have no competing interests.

## Authors' contributions

YOZ performed all experiments. YOZ and KL wrote the manuscript. TB contributed to the writing of the manuscript. All authors conceived the study. All authors read, commented and approved the final manuscript.
